# Children, Adolescents and Urine Hydration Indices—A Systematic Literature Review on Athletes and Non-Athletes

**DOI:** 10.3390/children12020171

**Published:** 2025-01-29

**Authors:** Georgios Papaoikonomou, Kyriaki Apergi, Olga Malisova

**Affiliations:** Department of Food Science and Technology, University of Patras, G Seferi 2, 30100 Agrinio, Greece; g_papaoikonomou@upatras.gr (G.P.); kyapergi@upatras.gr (K.A.)

**Keywords:** hydration, indices, athletes, non-athletes, children, adolescents

## Abstract

Background/Objectives: The importance of adequate hydration in children and adolescents has raised significant attention, both for its health benefits and for its role in supporting cognitive and physical performance. This is particularly important for young athletes who have increased dehydration risk due to high sweat loss and often inadequate water intake. The aim of this review is to systematically assess the hydration status of children and adolescents, including athletes, and to identify notable differences in hydration levels between these groups. Methods: A systematic literature search was conducted following PRISMA guidelines. PubMed, Scopus, and Scholar were searched for articles published between 2004 and 2024 on hydration in children and adolescents, focusing on urinary biomarkers such as urine osmolality, urine specific gravity, and urine color. Two independent reviewers screened the studies, and clinical studies or those involving chronic diseases, disabilities, or missing data were excluded. Results: Fifty-one articles met the inclusion criteria and were categorized into competitive athletes (*n* = 26) and non-athletes (*n* = 25). The review revealed that both athletes and non-athletes were frequently dehydrated. Among athletes, 81% of studies reported dehydration, while 69% of studies observed dehydration in non-athlete children. Biomarkers consistently indicated failure to meet recommended hydration guidelines in both groups. Conclusions: Despite existing hydration guidelines, dehydration is prevalent among children and adolescents, irrespective of athletic involvement. This underscores the urgent need for targeted interventions to improve hydration education and practices in schools, homes, and extracurricular settings.

## 1. Introduction

Water is an essential nutrient for humans, playing a crucial role in vital body functions [1]. A water loss of approximately 2% body weight can result in dehydration and be accompanied by a reduction in the efficiency of the body’s normal physiological processes, while further water loss of about 3% increases the risk of developing muscle cramps and can even result in heat stroke [2].

Both in children and adolescents, as well as in adults, there are many mechanisms to ensure that Total Body Water (TBW) stays within an age-specific range. However, TBW in infants is approximately 75%, declining gradually with age to reach approximately 55% in adolescents and adults [3].

The excretion of fluids from the human body occurs through four principal mechanisms: urination, defecation, respiration, and sweating. Fluid intake is mainly through water content from food and beverages, including water [4,5]. During exercise, thermoregulation is primarily achieved through the evaporation of sweat, which also results in water loss [6]. An average 70 kg athlete carries approximately 42 L of water in his body, and muscles contain approximately 75% of that water.

A small fluid imbalance of 2–3% of body weight immediately decreases athletic performance and reduces ability in training and competition conditions [7]. For this reason, proper hydration in younger ages is rather important. In children, it was observed that there was an overall increase in the rate of sweating per gland. At the same time, due to the lack of body fluids in combination with intense exercise, the mechanism of sweat glands is impaired, resulting in an undesirable increase in core temperature [8]. Thus, ensuring water balance in children and adolescents who engage in regular sports and participate in official sports competition is crucial, as the majority of children and adolescent athletes arrive at training sessions in a dehydrated state, which poses a significant health risk [6]. It is becoming more and more evident that achieving a state of adequate hydration is an ongoing process, with an indicator of the daily hydration state both in athletes and in non-athletes being thirst and the presence of dark-colored urine [9]. In the current literature, the most common urine hydration indices used to assess hydration from a urine spot are urine osmolality (UOsm), urine specific gravity (USG), and urine color (Ucol) [10].

According to research, most athletes should maintain UOsm less than 700 mOsm/kg, USG less than 1.020 g/mL, and Ucol pale-yellow (the color of lemonade, 1–3 on the Ucol Chart) [10]. For children, the recommendation of urine concentration, specifically a UOsm of ≥800 mmol kg as a marker of hypohydration in situations where exercise-induced dehydration is not a factor, has been made by numerous investigations. Furthermore, Ucol could be a good self-assessment method for evaluating hydration status in children and adolescents [11].

The period of childhood and adolescence, including athletes and non-athletes alike, represents a critical age when habits and dietary choices, including fluids, are largely determined [12]. Despite the current guidelines from the European Food Safety Authority (EFSA) [4] and the Institute of Medicine (IOM) [5], the majority of children and adolescent athletes and non-athletes are found to be in a state of dehydration, either due to the type of fluid consumed, access to fluids, or environmental factors and training protocols [13]. Moreover, the precise degree of dehydration that is detrimental to the physical performance and well-being of young athletes remains unclear [14], and research on dehydration in younger athletes is even more difficult, as there are many difficulties in assessing hydration state and specific levels of exercise [15].

Despite the recognition of hydration’s importance, there is a lack of comprehensive understanding regarding the hydration status of children and adolescents, and potential differences between athletes and non-athletes remain underexplored. This review addresses these gaps by synthesizing current evidence on hydration status in these populations, including both athletes and non-athletes, by examining urinary biomarkers.

## 2. Materials and Methods

### 2.1. Search Strategy

A systematic literature search was conducted following PRISMA guidelines [16] on the 27th of November 2024 using the PubMed, Scopus, and Scholar electronic databases. Primary outcomes were hydration status assessed by urinary biomarkers UOsm, USG, and Ucol, while secondary outcomes were concerned with the proportion of participants that were dehydrated. To systematically review the relevant literature, we employed a search strategy in PubMed and Scopus, utilizing a combination of keywords as follows: adolescent* OR youth* OR child* OR student* AND “physical activity” OR player* OR athletes OR “hydration status” OR dehydration. In Scholar, we used the same words but separately, since it does not support the same process as the above databases. No funding was received for this study and this review was not registered.

### 2.2. Selection Procedure

Duplicates of records found in the search were removed. Two independent researchers (GP and KA) screened the titles and abstracts of the remaining records to identify studies meeting the inclusion criteria. Any disagreements arising at this stage were resolved through discussion or consultation with a third reviewer. Full-text screening was performed by the same researchers and any disagreements were resolved similarly. No automation tools were used in the process. Review authors declare no competing interests.

### 2.3. Inclusions and Exclusions

The criteria for including the articles for this review were the following: (1) studies with children and adolescents (8–18 years) as participants. Although some of the eligible studies included younger ages, we decided not to include them as it was not part of the main objective of the review (weak memory, no available literature about hydration status on these age group); (2) studies assessing hydration status in athletes or non-athletes including hydration biomarkers such as Ucol, UOsm, and USG; (3) studies including children and adolescents engaged in regular sports activities or children without intense physical activity in schools and sports facilities; (4) articles published between 2004 and 2024. 

Studies were excluded if they were (1) clinical studies; (2) focused on chronic diseases or disabilities; or (3) grey literature.

**Figure 1:**
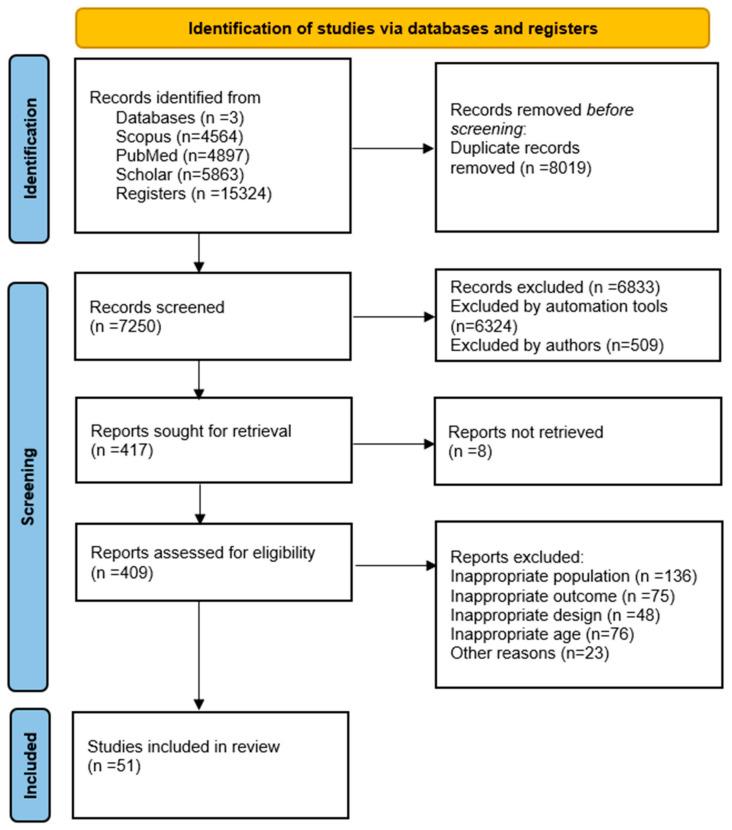
Flow chart for the search strategy.

Figure 1 presents the selection process. Of the 7250 studies screened, only 51 were found to be relevant for the final review. The remaining 51 articles were divided into 2 subcategories: (1) studies on athletes with hydration biomarkers (*n*= 26), (2) studies on non-athletes with hydration biomarkers (*n* = 25). Finally, the hydration indicators were categorized and divided according to their assessment of hydration.

### 2.4. Data Extraction

Standardized data extraction was carried out by the study team. All data were examined by one of the researchers (KA). The data were as follows: year, authors, article title, age group, sample size, study design, aim of the study, tools used for the assessment for physical activity or biomarkers (i.e., USG, UOsm), and hydration outcome summary. In the presentation of results, mean and standard deviation (SD) of urinary biomarker values (UOsm, USG, Ucol) and proportion (%) of participants classified as dehydrated based on established thresholds (e.g., UOsm ≥ 800 mOsm/kg, USG ≥ 1.020, Ucol ≥ 4) were used to summarize hydration status in each group. If studies reported missing or incomplete summary statistics (e.g., means, standard deviations) and sufficient data could not be extracted from the publication, no further action was taken to obtain or impute the missing information. Such studies were excluded from the synthesis but were mentioned in the systematic review narrative if relevant. No sensitivity analyses were conducted in this review as all included studies met the eligibility criteria and provided sufficient data for synthesis.

### 2.5. Classification of the Terms That Included in This Review

The following definitions have been established by EFSA [4] and IOM [5] to explore possible causes of heterogeneity among study results regarding the definition of hydration.

### 2.6. Hydration Biomarkers

Hydration status is the presumed state of healthy individuals who can maintain their water balance. Dehydration is the loss of water from the body, and hypohydration is the decreased water in the body [15]. USG and UOsm examine urine concentration, are strongly correlated, and increase with dehydration [17], while Ucol has recently displayed a positive relationship as a predictor of UOsm [11].

## 3. Results

Out of the initial 409 articles on fluid intake and hydration status in children and adolescents, both athletes and non-athletes, 51 were selected for this review and categorized into 2 subgroups: competitive athletes assessed hydration status (*n* = 26) and non-athlete children and adolescents assessed hydration status (*n* = 25). Εach study was compared to the cut off values of adequate hydration (UOsm < 700 mOsm/kg, USG < 1.020 g/mL, Ucol 1–3 values according color chart); upper arrow ↑ indicates adequate hydration and lower arrow ↓ indicates dehydration. In the first subgroup of athletes (*n* = 26), there were a variety in the methods used for hydration assessment. Specifically, nine articles used USG as a biomarker [13,18,19,20,21,22,23,24,25], two articles used UOsm [26,27], one article used Ucol [28], eight articles used two biomarkers (UOsm + USG or UOsm + Ucol or Ucol + USG) [29,30,31,32,33,34,35,36], and six articles used three biomarkers (UOsm, USG, Ucol) [37,38,39,40,41,42] (Table 1). In the second subgroup (*n* = 25), 24 studies used combinations of biomarkers (USG, UOsm, Ucol) [11,43,44,45,46,47,48,49,50,51,52,53,54,55,56,57,58,59,60,61,62,63,64,65] and free water reserve (FWR).

USG

Regarding Table 1, 26 studies focus on children and adolescents engaged in regular sports activities. Biomarkers such as UOsm, Ucol, and USG have been employed to assess hydration status. Specifically, in an intervention study, no statistically significant differences were observed in USG values between adolescents who consumed only water during exercise and participants who consumed a carbohydrate-electrolyte drink [18]. A study in Turkey found that 81% of the children pre- and post-exercise appeared dehydrated [19], while another study, assessing USG for 5 consecutive days in 13 adolescents, reported values above 1.022 g/mL [20]. Bergeron et al. found that pre-exercise hydration status in 8 adolescents according to the USG values was 1.025 g/mL, with a mean sweat loss of 1.9 L in single and dual sessions [21]; these findings were similar to a study by Gordon et al. in South Africa, which included 79 adolescents (USG 1.023 g/mL) [22]. In a study from Scotland [23], researchers observed that over three consecutive training days, 77% of 14 adolescent athletes had USG values exceeding 1.020 g/mL on days 1 and 3, while on day 2, 62% exceeded this threshold. In a Malaysian school, female athletes were found to have higher USG values (1.023 g/mL) compared to male athletes (1.020 g/mL) [24]. During competitions in Spain, the mean USG value of 306 adolescent athletes was reported as 1.019 g/mL [25]. Finally, in a three-day intervention study conducted in the USA involving 41 adolescents, baseline USG values decreased from 1.026 g/mL to 1.017 g/mL following hydration-focused educational lessons [13], showing that this is a validated approach for children [17].

Ucol

Only one study investigated hydration status using Ucol. Before exercise, the Ucol was measured using a 3-point scale. After exercise, the average score was greater than 4 according to the color scale chart. Regarding hydration knowledge, the average score was 16.25 from a total of 30 points [28].

UOsm

Regarding UOsm, it was observed that, on average, 63 adolescent players arrived for exercise in a state of mild hypohydration, with a mean UOsm of 830 ± 296 mOsm/kg. Among 36 children from Portugal, the average UOsm was 708.1 ± 175.4 mOsm/kg, with a positive free water reserve (FWR) of 83.2 ± 574.5 mL/24 h [26].

Threefold assessment

Six studies assessed athletes’ hydration status using a combination of methods, which appeared more valid than other approaches [12]. In Turkey, 16 adolescents demonstrated a mean UOsm of 989 mOsm/kg, a USG of 1.017 g/mL, and a Ucol score of 4 ± 1 based on six non-consecutive measurements [38]. In a five-day observational study in the USA, 33 children and adolescents showed an average UOsm of 796 mOsm/kg. Notably, their morning Ucol on the fourth day was statistically significantly lower than on other days [39]. Another observational study reported that adolescents had a mean UOsm of 881 ± 285 mOsm/kg before practice, with a USG range of 1.021–1.025 g/mL and Ucol values between 4 and 6 [27]. In a Turkish study involving 26 adolescents, Ucol was recorded as 3 ± 1 on the Ucol chart, USG as 1.021 g/mL, and osmolality as 903 ± 133 mOsm/kg, indicating severe dehydration during exercise [41]. Finally, a study using the Hydration Habits and Awareness Questionnaire to evaluate 67 children and adolescents found elevated UOsm levels (880 ± 261 mOsm/kg). Additionally, 53% of football players exhibited severe dehydration, as evidenced by USG values above 1.025 g/mL and an average Ucol of 5 [42].

Double biomarkers examination

Nine studies examined the hydration status of athletes using combinations of two biomarkers. In the UK, 21 adolescent athletes demonstrated a pre-exercise UOsm of 1319 ± 525 mOsm/kg [29]. In Puerto Rico, 24 adolescent athletes showed a 24-h USG of 1.028 g/mL, with 9 of them experiencing body mass losses exceeding 2% [30]. In Greece, 107 children and adolescents were assessed using Ucol and USG. Based on the Ucol chart, 100 participants were classified as dehydrated, and pre-exercise USG values exceeded 1.031 g/mL [31]. An observational study found that 85% of 35 adolescents were dehydrated, with Ucol scores greater than 5 and USG values exceeding 1.020 g/mL [32]. Arnaoutis et al. [33] reported that 95 of the 107 children and adolescents were dehydrated based on morning urine analysis. In a follow-up study in 2015, 76.3% of 59 adolescents were found to be dehydrated both before and after exercise [34]. In Singapore, 105 adolescent athletes had a mean Ucol of 3.7 on the Ucol scale (33), and 67% of 46 children and adolescents exhibited UOsm values above 700 mOsm/kg [36]. Table 2 provides information on children and adolescents who are not athletes, and for whom hydration status was assessed using a variety of methods, including Ucol, UOsm, USG, and any changes in body weight before and after assessment.

USG

Regarding USG, 90% of 141 adolescents had values greater than 1020 g/mL [43], while the mean USG value in 68 children and adolescents was 1.020 ± 0.011 g/mL [44].

Ucol

Based on the Ucol scale of 1–8 [2], 59.6% of children and adolescents were categorized as dehydrated and only one third (33.0%) were found to be well hydrated [45].

UOsm

Sixteen studies examined hydration status via UOsm. The lowest value was observed at 570 mOsm/kg [46], and the highest at the extreme value of 1270 mOsm/kg in 168 Italian children during school hours [50]. In Belgium, at the start of school, UOsm in 371 children was over 888 mOsm/kg [58], while in Zaragoza, Spain, mean first morning UOsm was above 800 mOsm/kg [56]. Among 519 children in Egypt aged 9–11, UOsm was 819 mOsm/kg [53]. In France, more than 519 children had a UOsm between 801 and 1000 mOsm/kg [52]. Bar-David et al. twice examined voluntary hydration in 58 and 429 children of Israeli origin and found UOsm of 856 and 883 mOsm/kg, respectively [48,49]. In 242 children aged 7–12 years, mean 24-h UOsm was 804.7 mOsm/kg [47], while a mean 24-h osmolality (667 ± 158 mOsm/kg) was observed in 172 students in Portugal [57]. A large-scale study in the USA by Kenney et al. [54] involving 4134 children and adolescents aged 6–19 years found that the mean UOsm across the population was 755.5 mOsm/kg. In the same country, 63% and 66% of 548 children aged 9–11 in LA and NYC, respectively, had elevated UOsm (800 mOsm/kg). Similar results were found in Egypt and Poland with values of 899 and more than 1000 mOsm/kg, respectively [60,61]. Most children and adolescents have a UOsm greater than 800 mOsm/kg) with the exception of two studies [46,54] (570 mOsm/kg and 755.5 mOsm/kg, respectively).

Twofold and threefold assessment

Five studies examined hydration status in children and adolescents using two or three biomarkers to increase the validity of their results [6]. In 210 children and adolescents from Greece, 24-h osmolality and 24-h USG were observed to be 686 mOsm/kg and 1.018 g/mL, respectively [62]. Similarly, in 150 children, again from Greece, 24-h osmolality and USG were 707 mOsm/kg and 1019 g/mL, respectively [64]. In addition, the same researcher, examining 210 children and adolescents, observed that the mean Ucol value was 3 ± 1 and the osmolality was 686 mOsm/kg, suggesting that Ucol displayed a positive relationship as a predictor of UOsm [11]. In 75 children in an intervention study in the USA, urine sample analysis was conducted on all three biomarkers (UOsm, Ucol, USG). For low-intervention subjects, the mean value of UOsm was 790 mOsm/kg, USG was 1.023 g/mL, and Ucol was 6, while in high-intervention subjects, the values were statistically significantly decreased *p* < 0.01 [65].

The included studies employed diverse methodologies, including different urinary biomarkers (UOsm, USG, Ucol) and thresholds for defining dehydration, which may have contributed to heterogeneity in results. No sensitivity analyses were conducted in this review. All included studies met eligibility criteria, and no imputed or incomplete data were used in the synthesis. While the risk of bias due to missing results was considered low overall, the lack of data stratification in some studies might limit subgroup analyses’ robustness. No evidence suggested selective outcome reporting or consistent omissions of null findings.

## 4. Discussion

In childhood and adolescence, maintaining adequate hydration levels is crucial for promoting health, supporting proper development, enhancing clarity, and ensuring that the body functions normally. Given that increased sweat rhythm and intense breathing in this age group can lead to a more rapid loss of body fluids, it is of particular importance to ensure adequate hydration [39]. The objective of the present review was to focus on their participation in sports as athletes. This systematic review specifically aimed to examine hydration behaviors and biomarkers in relation to physical activity and its impact on fluid balance. Our findings indicate that both athletes and non-athletes are commonly in a state of dehydration. This suggests that inadequate hydration is a widespread issue, not only for children engaged in organized sports but also for those who may be less active but still vulnerable to dehydration due to environmental factors, lifestyle habits, or insufficient fluid intake.

Assessing hydration status in children and adolescents poses significant challenges due to the lack of a definitive ’gold standard’ methodology tailored for assessing hydration status in this age group [66]. Unlike adults, whose hydration markers are relatively well-defined, the physiological characteristics of children and adolescents require more nuanced approaches to accurately gauge their hydration levels. To address this, some studies have employed a combination of biomarkers to enhance the precision and reliability of their findings. By integrating multiple biomarkers, researchers can obtain a more comprehensive understanding of hydration status, reducing the potential limitations that may arise when relying on a single marker [9].

However, it is important to note that while biomarkers such as USG, Ucol, and UOsm provide useful screening tools for assessing hydration status, they should be complemented with other assessment methods for a more comprehensive approach. Monitoring fluid intake, environmental conditions, and physical activity levels is essential to ensure accurate hydration management in young athletes. These factors can influence hydration status and may provide additional context for interpreting biomarker data. For example, higher temperatures and intense physical activity can increase fluid loss, necessitating greater hydration efforts [10].

In adult athletes, a USG below 1.020, pale-yellow Ucol (1–3 on the Ucol scale), and a UOsm below 700 mOsm/kg are considered as a typical indication of a well-hydrated status [10]. These hydration markers can also be applied to adolescents, as the European Food Safety Authority (EFSA) recommends the same daily water intake of 2.500 mL for teenagers aged 14–18 years [67]. However, hydration needs in adolescents may vary depending on factors such as activity levels, body size, and climate. Thus, while these markers offer useful benchmarks, it is crucial to tailor hydration strategies to individual needs to ensure optimal health and performance. This review revealed that the majority of studies examining biomarkers in athletes and non-athletes did not find the expected guidelines for optimal hydration levels. The findings underscore the importance of prioritizing hydration assessment for both athletes and students, as proper hydration is critical for maintaining health, cognitive clarity, and physical performance. There is an urgent need for healthcare practitioners and educators to raise awareness about the significance of achieving and maintaining adequate fluid intake.

Studies utilizing urinary biomarkers as a method of assessment reveal that participants were not only inadequately hydrated but also lacked sufficient knowledge about proper hydration practices. This trend was observed in both athletes and non-athletes. For example, in a study of 70 adolescent athletes across various sports, the mean USG was 1.020 ± 0.006 g/mL for males and 1.023 ± 0.009 g/mL for females, both exceeding the recommended threshold of 1.020 g/mL [24]. Additionally, 86.9% of participants in the study reported that they did not perceive thirst as a late indicator of dehydration, further emphasizing the gap in hydration awareness [24]. When examining hydration, weather conditions are a rather important factor. In 2 studies, with 58 adolescents and 429 children in Israel, respectively, where temperature ranges between 35–37 °C, very high morning UOsm (mean values: 856 ± 232 mOsm/kg for adolescents and 883 ± 201 mOsm/kg for children) was found [48,49]. Similar results were found in Cyprus, where 90% of 141 adolescents, at comparable temperature conditions, had USG above 1020 g/mL [43]. In Egypt, the mean UOsm was 899 ± 59.4 mOsm/kg in 180 children [60]. These findings highlight the need for proper awareness and nutritional education in these age groups under such weather conditions.

The feeling of thirst was found not to be a reliable indicator for adequate water intake. An observational study involving thirty-three twelve-year-old athletes utilized both urinary biomarkers and a ten-point Likert scale questionnaire to assess hydration. The study found a strong correlation between USG and UOsm (r = 0.964, *p* < 0.001); however, measurements of hydration status did not align with the athletes’ perceived thirst sensation. [30]. This phenomenon could be attributed to the young age of the participants, since there is existing evidence supporting that the physiology of hydration and thirst signals may not be fully developed in children and adolescents [68].

It has been proved that thirst is not often a good indicator of dehydration in exercise, as athletes can lose as much as 1.5 L of fluid before feeling thirsty [69]. Therefore, relying on thirst to guide fluid intake during exercise is insufficient to prevent further dehydration [33]. Voluntary fluid intake (Ad Libitum) or drinking to satisfy thirst was found to replenish, at most, two-thirds of sweat loss that occurs during exercise [6]. It is important to note that these two phrases are not synonymous. “Ad libitum” refers to a state of consumption that is not influenced by external factors and can occur at any time and in any quantity. In contrast, the phrase “drinking to satisfy thirst” describes the process by which the thirst mechanism leads to the consumption of fluids [8]. 

Findings in adolescent athletes regarding fluid losses during aquatic sports declare significantly reduced sweat losses in senior elite-level swimmers and water polo players when compared to non-aquatic team sports [70]. A cross-sectional study with 107 thirteen-year-old children observed that 88.7% exhibited signs of dehydration. Furthermore, no significant difference in urine indices with fluid deficits (USG > 1.020, Ucol > 5) were observed between two groups of swimmers, namely 35 professional swimmers and 41 less active swimmers [32]. This indicates that athletes may experience dehydration more acutely due to the increased intensity, suggesting that coaches and health experts must pay close attention to the need for proper hydration monitoring.

Ucol is a reliable indicator of hydration, as shown in a study of 210 children aged 8–14 years from Greece, where Ucol was positively correlated with UOsm (: 0.45, *p* < 0.001) [11]. This method can be used by experts or through self-assessment using a Ucol chart in everyday practice [2]. Implementing this in educational structures can enhance children’s knowledge of hydration and serve as a practical marker of hydration status. Especially for athletes, it is important to assess hydration levels, either by self-assessment or by calculating individual sweat rates, which vary from less than 500 mL/h to more than 2 L/h for inactive people and between 3 and 5 L/h for active individuals [10].

Proper hydration needs an ongoing educational process, and this will require highly trained coaches, regular hydration classes, Ucol charts, and freely available water bottles on the field [13]. Regarding sugar-sweetened beverages (SSBs), there is a significant concern about their high consumption by children and adolescents, especially because high sugar content can increase daily energy intake, thereby increasing the risk of obesity and health related implications [71]. For instance, one study in Italy reported that obese children were less hydrated than normal-weight children using the Free Water Reserve method (FWR = median (IQR): 0.80 (−0.80–2.80) hg/day vs. 2.10 (0.10–4.45) hg/day, *p* < 0.02) [55]. For this reason, it is important to educate children and adolescents, as well as parents, to prefer plain water over other caloric beverages for hydration [72].

The studies reviewed highlighted that many athletes, despite their higher physical activity levels, are often unaware of their hydration status and do not drink enough to compensate for the fluid lost during exercise. This lack of awareness, combined with the physiological changes that occur during physical exertion, can lead to significant fluid deficits, which may impair athletic performance, delay recovery, and even increase the risk of heat-related illnesses. For non-athletes, particularly those in school environments, a lack of access to water, irregular fluid intake habits, and insufficient understanding of hydration needs contribute to chronic low hydration levels.

Strengths and limitations

The evidence from this review underscores the need for comprehensive hydration strategies that are tailored to the unique needs of both athletes and non-athletes in childhood and adolescence. Schools, sports organizations, and healthcare professionals should work together to implement hydration education and provide practical solutions to ensure that children and adolescents receive adequate fluids throughout the day, particularly during periods of increased physical activity. Future research should focus on further exploring the factors that influence hydration behaviors in young people, as well as developing targeted interventions to improve hydration knowledge and practices.

The present study is constrained by the absence of longitudinal and observational studies specifically examining children’s hydration behaviors within the school environment, which limits the ability to identify long-term patterns or trends in hydration status. Moreover, the lack of standardized protocols for measuring hydration status poses a challenge in ensuring consistency and comparability across studies. Variations in hydration recommendations across different regions, climates, and populations further complicate the interpretation and generalizability of findings, as the studies included in the review utilized various methodologies, incorporating different urinary biomarkers (UOsm, USG, Ucol) and varying thresholds to define dehydration, which may have introduced heterogeneity into the results. Additionally, self-reported data on hydration practices, while often necessary, may be subject to recall bias or social desirability bias, reducing the reliability of the data. Another limitation is the insufficient exploration of socioeconomic factors, such as access to clean drinking water, parental education, and household income, which can significantly influence hydration habits and may exacerbate disparities in health outcomes related to hydration. Limited stratification by key variables such as age, sex, ethnicity, or geographic region restricted the ability to draw subgroup-specific conclusions. While no formal tests for publication bias were conducted, the focus on published studies may have excluded null or non-significant findings, potentially overestimating dehydration prevalence. Finally, the lack of sensitivity analyses to evaluate the robustness of findings across various assumptions limits the ability to confirm the stability of the synthesized results.

Despite these limitations, this review is strengthened by a substantial body of research that examines hydration from diverse perspectives, including the use of objective biomarkers such as UOsm, USG, Ucol, and free water reserve. These methodologies provide robust tools to assess hydration status and offer valuable insights into both acute and chronic hydration levels. Additionally, this review is pioneering in its attempt to differentiate hydration status between children and adolescents who engage in regular exercise and those who limit their physical activity to standard school-based play. This distinction is critical, as exercise increases fluid losses through sweating and respiratory processes, potentially placing more active children at greater risk of dehydration. By addressing this gap, the study contributes to a nuanced understanding of hydration behaviors in youth and lays the groundwork for tailored hydration recommendations based on activity levels and age.

## 5. Conclusions

Ensuring adequate hydration during childhood and adolescence is essential due to the high levels of physical activity and the physiological demands characteristic of this developmental period. This review, involving childhood and adolescent athletes and non- athletes, revealed that despite existing guidelines and expert recommendations, many children and adolescents, both athletes and non-athletes, remain in a state of dehydration, as they did not manage to meet the recommended values for adequate hydration. Specifically, among athletes, 81% of studies reported dehydration, while 69% of studies observed dehydration in non-athlete children. Contributing factors may include the type of fluid consumed, accessibility to water, environmental conditions, and training protocols. Future research should focus on developing standardized protocols for hydration assessment and conducting longitudinal studies to explore the long-term impacts of hydration behaviors in children and adolescents. Furthermore, it is essential to consider the role of environmental, cultural, and socioeconomic factors to better inform targeted interventions that promote equitable access to hydration resources and education to this age group.

## Figures and Tables

**Table 1:** Summary of data by urine biomarker assessment for children and adolescent athlete’s group.

Year & Author	Aim of the Study	Study Design	Sample Size (*n*) and Age Group Target	Country	Biomarkers Used	Desirable Values ^1^	Assessment Tools	Hydration Outcome Summary
[18]	Ad libitum fluid intake, vs. a 6% carbohydrate/electrolyte drink	Randomised crossover	14 adolescents	USA	USG	↓	Assess pre-exercise hydration status	Pre-exercise USG: water group: mean: 1.025 g/mL (SD: 0.005), CHO-E group 1.024 g/mL (0.006) *p* > 0.05.
[19]	Fluid intake, hydration status and body weight changes.	Observational study	22 children	Turkey	USG	↓	Hydration status and weight before and immediately after the training.	81.2% were dehydrated. USG Morning: 1.022 g/mL (0.005), USG Pre-training: 1.023 g/mL(0.004), USG Post-training: 1.024 g/mL (0.004).
[20]	Determine risks of fluid deficits and drinking strategy.	observational study	13 adolescents	USA	USG	↓	Pre-practice (USG), and body weight changes in 5 days.	USG day 1: 1.024 (SD: 0.005), day 2: 1.023 (0.004), day 3: 1.024 (0.004), day 4: 1.022 (0.006), and day 5: 1.022 (0.003).
[21]	To examine pre- and post-play hydration status.	Observational study	8 adolescents	USA	USG	↓	Pre-exercise hydration status.	Pre-play USG: 1.025 g/mL (0.002); and total sweat loss (1.9 L (0.2)
[22]	Hydration status and fluid intake.	Observational study	79 adolescents	South Africa	USG	↓	A self-administered questionnaire.	USG 1.023 g/mL (SD: 0.006) before and after 1.024 g/mL (0.007) training. loss of body weight was 0.7 ± 0.7%.
[23]	Hydration status and fluid balance during three sessions.	Observational study	14 adolescents	Skotland	USG	↓	BM change, urine output volume fluid intake sweat rate sweat loss.	(USG > 1.020 g/mL; 77% on days 1 and 3, 62% on day 2). BM loss occurred in 1–3 (0.69 (0.22), 0.42 (0.25), and 0.38 (0.30) kg respectively, *p* < 0.05).
[24]	Hydration status, knowledge and fluid habit among athletes.	Observational study	70 adolescents	Malaysia	USG	↑	Hydration Knowledge and Hydration Habit Questionnaire	Results males USG 1.020 g/mL (0.006) females USG 1.023 g/mL (0.009) (*p* > 0.05).
[25]	Observation of water intake during match.	Observational study	306 adolescents	Spain	USG	↑	Water intake recorded and level of hydration was evaluated using USG	The mean urine density was 1.019 g/mL (SD: 0.007).
[13]	Effects of educational intervention (EI) on hydration practices	Intervention study	41 adolescents	USA	USG	↓	24-h fluid intake logs, and questionnaires. 24 h means fluid consumed (FC) and water consumed (WC)	USG baseline 21 subjects EI: 1.026 ± 0.006 g/mL; 20 subjects NI: 1.023 ± 0.009; *p* = 0.118, confidence interval CI 95% ([20.001 to 008]), improved 3-days post-EI (EI: 1.017 (0.010); NI: 1.026 (0.007); *p* = 0.004, CI 95% [20.015 to 20.003]
[28]	Hydration status of athletes before and after training.	observational study	16 adolescents	Indonesia	Ucol	↓	Questionnaire related to hydration.	Pre-Training 3.06. Post Training 4.06. The majority had a fair knowledge of hydration with an average score 16.25 point from total 30 points.
[26]	Evaluation of the hydration status.	observational study	36 children	Portugal	UOsm	↓	(FWR [mL/24 h] = urine volume [mL/24 was used to assess the hydration status.	Mean Osmolality (mosmol/L) 708,1 (SD 175.4) Urinary Volume (mL/24 h) 936.0 (408.3).
[37]	Examine hydration status	observational study	63 children	USA	UOsm	↓	Self-report diary. Fluid consumed ad libidum.	On average, players arrived mildly hypohydrated 830 mOsm/kg (SD: 296).
[38]	Assess the hydration status.	observational study	16 adolescents	Turkey	UOsm, Ucol, USG	↓	urinary measurements, beginning, 5 days later, one day before competition.	The average values for all samples were 989 mOsm/kg (SD: 205), USG 1.017 g/mL (0.010) for specific gravity and 4 + 1 units for color.
[39]	To measure hydration status.	observational study	33 children and adolescents	USA	UOsm, Ucol, USG	↓	Sweat rate, hydration score, thrist score.	UOsm mean 796 mOsm/L (293). USG values and UOsm were significantly correlated (r = 0.964, *p* < 0.001).
[27]	Thermoregulatory and hydration responses.	observational study	25 adolescents	USA	UOsm, Ucol, USG	↓	Sweat Rate, hydration knowledge questionnaire.	UOsm: 881 mOsm/L (SD: 285) before practices(BP); 856 (259) after practices(AP). USG: 1.021 g/mL to 1.25 g/mL (BP); 1.023 to 1.25 (AP). Ucol: 4 to 6 (BP); 4 to 6(AP).
[40]	To assess the hydration status.	cohort study	36 adolescents	USA	UOsm, Ucol, USG	↑	Educational Intervention volume of fluid consumed (Fvol).	UOsm Pre-control: 635 mOsm/L (146), Pre EI: 737 mOsm/L (153). Post-control: 706 mOsm/L (149), post-EI: 821 mOsm/L (152). USG. Pre-control:1.016 μg/L (SD: 0.004), Pre EI:1.017 μg/L (0.005). Post-control: 1.018 μg/L (0.004) Post EI:1.019 μg/L (0.005). Ucol: Pre-control:3.9 (0.7), Pre-EI: 4.1 (0.8) post-control: 4.2 (0.2). Post EI: 4.5 (0.8).
[41]	compare the hydration status with different methods.	observational study	26 adolescents	Turkey	UOsm, Ucol, USG	↓	USG was measured with 3 different methods.	3 (1) for color, 1.021 μg/L (4) for USG and 1.021 g/mL (3) for USG (strip) and 1.021 (4) for USG (refractometry), and 903 mOsm/kg (133) for UOsm.
[42]	To assess the hydration status.	Observational study	67 children and adolescents	USA	UOsm, Ucol, USG	↓	Hydration Awareness Questionnaire, Hydration Habits Questionnaire, Exit Questionnaire.	Baseline USG: [(1.022 g/mL (SD: 0.007)], UOsm [(880 mOsm/L (261)]. On average 56% of football adolescents and 53% of soccer boys experienced significant to severe dehydration (USG values ≥ 1.025). Average Ucol = 5.
[30]	Hydration status of athletes before, immediately after, and 24 h	Observational study	24 adolescents	Puerto Rico	USG, Ucol	↓	Body mass measured before, after, and 24 h after a training session.	The 24h USG was 1.028 g/mL (SD: 0.004) and 1.027 g/mL (0.005). Nine subjects lost ≥ 2% body mass.
[31]	Whether an intervention increasing fluid intake.	Interventional study	92 adolescents	Greece	USG, UOsm,	↓	Hydration status	Hydration status was improved significantly in the INT 61 subjects(intervention [USG: pre = 1.031 g/mL (SD: 0.09),post 1.023 g/mL (0.012) *p* < 0.05; UOsm: pre = 941 mOsm/kg (30), post = 782 mOsm/kg (34), *p* < 0.05].
[32]	Compare daily hydration profiles.	Observational study	35 adolescents	Australia	Ucol, USG	↓	Questionnaire	Fluid deficits (USG > l.020 g/mL. Ucol > 5) Hypohydration was present in 85% from the athlete group and 78% from the control group.
[33]	Assess pre-exercise hydration status.	Observational study	107 children and adolescents	Greece	Ucol, USG	↓	Body weight changes	88.7% (95 of 107) were hypohydrated based on first morning urine sample. According Ucol chart, 93.7% (100 of 107) were classified as hypohydrated.
[29]	Hydration Status, Fluid Intake, and Electrolyte Losses	Observational study	21 adolescents	UK	UOsm, Ucol	↓	Pretraining and posttraining measurements of body mass	Pretraining UOsm: 1319 mOsmol/kg (SD: 525), posttraining: 687 mOsmol/kg (389), decreased significantly during training (*n* = 19, *p* = 0.001, η² = 0.47)
[34]	Assess hydration status.	Observational study	59 adolescents	Greece	Ucol, USG	↓	First morning urine sample before and after practice and body weight was recorded.	76.3% were hypohydrated: USG values ≥ 1.020 g/mL, and Ucol scale: 5–6 (76.3%). The majority of the athletes dehydrated even more during practice despite fluid availability. 21% Pre-training USG in a hypohydrated state.
[35]	Hydration status	Observational study	105 adolescents	Singapore	Ucol, USG	↓	hydration status assessed upon rising, before a low and a high-intensity session	Overall, 105 Morning Ucol 3.7 (SD: 2.0). 20–44% of athletes were hypohydrated, with 21–44% and 15–34% of athletes commencing low- and high-intensity training in a hypohydrated state, respectively.
[36]	Hydration status	Observational study	46 children and adolescents	USA	UOsm, USG	↓	First morning urine sample.	67% of athletes were hypohydrated. UOsm > 700 mOsmol/kg. (before: 828 vs. after: 630 mOsmol/kg, *p* = 0.001.

^1^ Desirable values according to ACSM. USG less than 1.020 g/mL, a pale-yellow urine color, and UOsm less than 700 mOsm/kg. UOsm: urine osmolality, Ucol: urine color, USG: urine specific gravity. ↓ Not desirable, ↑ Desirable.

**Table 2:** Summary of data by urinary biomarker assessment for non-athlete’s children and adolescent group.

Year and Author	Aim of the Study	Study Design	Sample Size (*n*) and Age Group Target	Country	Biomarkers Used	Desirable Values ^1^	Assessment Tools	Hydration Outcome Summary
[43]	Assess hydration status and total water intake	οbservational	141 adolescents	Cyprus	USG	↓	Detailed food and fluid records.	Ninety percent (USG > 1.020). Seriously dehydrated students felt less alert in the morning (*p* < 0.035) feeling of thirst was similar between all groups.
[44]	USG, 24-h urine sample, body fat % and lean mass.	οbservational	68 childrenand adolescents.	USA	USG	↑	Three 24-h dietary recalls and analyzed	USG score was 1.020 g/m (SD: 0.011)
[45]	Fluid intake and hydration status.	οbservational	230 children and adolescents	Malaysia	Ucol	↓	15-item beverage intake questionnaire	Dehydrated (59.6%) and only one-third (33.0%) were well hydrated.
[46]	If FandV intake is improving hydration status.	Observational study	442 children	Germany	UOsm	↑	3-d weighed dietary records and 24-h urine collection	22% of children Negative FWR values, UOsm: Boys: 7–10 yrs 665 mOsm/L (520, 854). Girls: 7–10 yrs: 570 mOsm/L (440, 744).
[47]	Hydration status and dietary water intake.	οbservational	242 children	Spain	UOsm	↓	3-d weighed dietary records and 24-h urine collection	UOsm Total mean: 804.7 mOsm/kg (SD: 205.3)
[48]	Voluntary dehydration by morning and noon-time UOsm	Observational study	58 children	Israel	UOsm	↓	Urine sample	Mean morning UOsm was 856 mOsm/L (SD: 232). 620 mOsm/L (164) in the hydrated group and 997 mOsm/L (128) in the dehydrated group (F (1,49) = 83.78, *p* = O.OOO).
[49]	Voluntary dehydration school children.	Observational study	429 children	Israel	UOsm	↓	One urine sample for osmolality measurement	The mean UOsm was 883 mOsm/L (SD: 201) among boys and 844 mOsm/L (218) among girls (*p* = 0.057).
[50]	The effects of drinking water during school day.	Observational study	168 children	Italy	UOsm	↓	UOsm greater than 800 mOsm/L = dehydration	Voluntary dehydration at the beginning of the school (84%). The mean UOsm at the beginning of the school day was 1270 mOsm/L (SD: 281).
[51]	Hydration status of children in the USA.	Observational study	548 children	USA	UOsm	↓	Diet record water intake and Self collected a urine sample	Elevated UOsm (>800 mmol/kg) was observed in 63% and 66% of participants in LA and NYC, respectively.
[52]	Assess the morning hydration status.	Observational study	529 children	France	UOsm	↓	Questionnaire on fluid and food intake at breakfast	More than a third of the children had a UOsm between 801 and 1000 mosm/kg while 22.7% had a UOsm over 1000 mosm/kg.
[53]	The prevalence of morning mild hydration deficit.	Observational study	519 children	Egypt	UOsm	↓	Questionnaire on breakfast intakes	The mean UOsm of children was 814 mOsmol/kg:(57%) and >1000 mOsmol/kg (24.7%). 63% of the children skipped breakfast.
[54]	Hydration status of US children and adolescents.	Observational study	4134 Children and Adolescents	USA	UOsm	↓	Urine collection	The mean UOsm across the population was 755.5 mOsmol/kg (range = 34–1394; SE = 7.4.
[55]	hydration status between obese and normal children.	Observational study	175 Children	Italy	UOsm	↑	3 day weight dietary	UOsm: Obese: 741 mOsm/kg (589–908), Normal: 645 mOsm/kg (491–836) *p* < 0.05.
[56]	Hydration status	Observational study	194 Adolescents	Spain	UOsm	↓	Only one 24-h dietary recall	Mean first morning UOsm was above 800 mOsm/kg in both groups.
[57]	Evaluation of the hydration status.	Observational study	172 Children	Portugal	UOsm	↑	24 h food recall and 24 h urine collection.	Mean UOsm 708,1 mosmol/l (SD: 175,4). Urinary Volume (mL/24 h) 936,0 (408,3). FWR (mL/24 h) 83,2 (574,5).
[58]	To examine children’s hydration status at school.	Observational study	371 Children	Belgium	UOsm	↓	FFQ	Mean UOsm of 888 mosmol/kg (SD: 192) was found in the school-start sample and 767 mosmol/kg (310) in the school-day sample.
[59]	To evaluate the prevalence of voluntary dehydration.	Observational study	475 Children	Brazil	UOsm	↓	Weight/height measurements	Voluntary dehydration occurred in 63.2% of the students and was more frequent in males than in females.
[60]	Assess the prevalence of dehydration among school children.	Intervention	180 Children	Egypt	UOsm	↓	Urine osmolality was tested to the students	68% of the students were dehydrated and significantly decreased after health education to reach 47.8%. mean UOsm was 899 mosm/kg (SD: 59.4) in dehydrated and 412 (199) mosm/kg in the hydrated students.
[66]	Association between hydration status and body composition.	Observational study	264 Children and Adolescents	Poland	UOsm	↓	Urine sample given during school stay (after breakfast)	Improper hydration was found in 53% of children, and 16.3% of them were severely dehydrated during a school day (UOsm > 1000 mosm/kg.
[62]	Examine hydration–by 24-h urine osmolality.	Observantional study	210 Children and Adolescents	Greece	UOsm USG	↑	Fluid intake for 2 days	Total 24-h USG 1.018 g/m (SD: 0.006), Total 24-h Urine volume (mL) 1335 (620).
[63]	Hydration status and dietary behaviour	Observantional study	717 Children	Germany	FWR	↑	24-h urine and 3-day weighed food records	FWR (mL/day) 189 boys 4–7 yrs 11 (−64, 130), 173 boys 9–11 yrs, −14 (−89, 166) 181 girls 4–7 yrs 60 (−22, 175), 174 girls 9–11 yrs 111 (−23, 279).
[64]	Fluid intake and urinary hydration markers	Observantional study	150 Children	Greece	UOsm USG	↑	Fluid intake for 2 days. 24-h urine collection	24-h [UOsm boys 777 mmol/kg(226) girls 637(200). USG boys 1021 g/m (0.006) girls 1017 (0.005). 24-h Uvol (mL): boys1268 (561) girls 1307 (577)].
[11]	Ucol is a practical tool for hydration assessment.	Observantional study	210 Children and Adolescents	Greece	UOsm Ucol	↑	Urine sample first morning and before lunch) and 24-h sampling.	Mean Ucol was 3 ± 1 and UOsm 686 mmol/kg (SD: 223) UCol displayed a positive relationship as a predictor of UOsm (R²: 0.45, *p* < 0.001).
[65]	Effects of water intake on urinary markers of hydration.	Intervention	75 Children	USA	Uomso Ucol USG	↓	Ad libidum and restricted or high consume water intake	UOsm.low 20 subjects: 912 mOsmol/kg(199), AdL 23 subjects: 790 mOsmol/kg(257). High 32 subjects: 260 mOsmol/kg (115) and USG [low: 1.023 g/mL (0.005), AL: 1.020 g/mL (0.007), high: 1.005 g/mL (0.004)
[67]	Hydration status on body weight.	Observational study	372 children and adolescents	Spain	Urine and blood markers	↓	Hydration Status Questionnaire Adolescent-Youth (HSQ-AY)	According to BMI, overweight/obese individuals consumed less water than normal weight ones.

UOsm: urine osmolality, Ucol: urine color, USG: urine specific gravity: ^1^ Desirable values according to ACSM. USG less than 1.020 g/mL, a pale-yellow urine color, and UOsm less than 700 mOsm/kg. ↓ Not desirable, ↑ Desirable.

## Data Availability

The original data presented in the study are openly available in PubMed.

## Abbreviations

The following abbreviations are used in this manuscript:

UOsm	Urine Osmolality
USG	Urine Specific Gravity
Ucol	Urine Color
